# TGR5 supresses cGAS/STING pathway by inhibiting GRP75-mediated endoplasmic reticulum-mitochondrial coupling in diabetic retinopathy

**DOI:** 10.1038/s41419-023-06111-5

**Published:** 2023-09-01

**Authors:** Yan Li, Lingpeng Zhu, Meng-Xia Cai, Zi-Li Wang, Miao Zhuang, Cheng-Ye Tan, Tian-Hua Xie, Yong Yao, Ting-Ting Wei

**Affiliations:** 1 Department of Ophthalmology, The Affiliated Wuxi People’s Hospital of Nanjing Medical University, Wuxi, 214023 P. R. China; 2 Center of Clinical Research, The Affiliated Wuxi People’s Hospital of Nanjing Medical University, Wuxi, 214023 P. R. China

**Keywords:** Mechanisms of disease, Calcium signalling

## Abstract

Diabetic retinopathy (DR) is a serious and relatively under-recognized complication of diabetes. Müller glial cells extend throughout the retina and play vital roles in maintaining retinal homeostasis. Previous studies have demonstrated that TGR5, a member of the bile acid-activated GPCR family, could ameliorate DR. However, the role of TGR5 in regulating Müller cell function and the underlying mechanism remains to be ascertained. To address this, high glucose (HG)-treated human Müller cells and streptozotocin-treated Sprague-Dawley rats were used in the study. The IP3R1-GRP75-VDAC1 axis and mitochondrial function were assessed after TGR5 ablation or agonism. Cytosolic mitochondrial DNA (mtDNA)-mediated cGAS-STING activation was performed. The key markers of retinal vascular leakage, apoptosis, and inflammation were examined. We found that mitochondrial Ca^2+^ overload and mitochondrial dysfunction were alleviated by TGR5 agonist. Mechanically, TGR5 blocked the IP3R1-GRP75-VDAC1 axis mediated Ca^2+^ efflux from the endoplasmic reticulum into mitochondria under diabetic condition. Mitochondrial Ca^2+^ overload led to the opening of the mitochondrial permeability transition pore and the release of mitochondrial DNA (mtDNA) into the cytosol. Cytoplasmic mtDNA bound to cGAS and upregulated 2’3’ cyclic GMP-AMP. Consequently, STING-mediated inflammatory responses were activated. TGR5 agonist prevented retinal injury, whereas knockdown of TGR5 exacerbated retinal damage in DR rats, which was rescued by the STING inhibitor. Based on the above results, we propose that TGR5 might be a novel therapeutic target for the treatment of DR.

## Introduction

Diabetic retinopathy (DR) is one of the microvascular complications of diabetes mellitus (DM) and the leading cause of visual impairment among the working population [1]. The number of diabetes cases was estimated at 451 million in 2017 and is projected to reach 693 million by 2045 [2]. Almost all people with type 1 diabetes and about 60% of people with type 2 diabetes are anticipated to develop DR within the next two decades [3]. However, the pathogenesis of DR is complex, and only a few safe and effective treatments are available for patients with DR.

In the past decades, research on DR has focused on neovascularization. Long-term injection of anti-vascular endothelial growth factor (anti-VEGF) is one of the effective treatments for DR in clinics [4]. Nevertheless, evidence now shows that Müller cell dysfunction is a key player in DR progression. Müller cells cross the entire retina and interact with the retinal microvasculature to promote communication between blood vessels and neurons. Under normal conditions, Müller cells secrete factors such as PEDF and GDNF to help maintain the integrity of the blood-retinal barrier (BRB). However, they release VEGF and tumor necrosis factor (TNF) to improve vascular permeability under pathological conditions such as hypoxia. Besides, together with neurons, endothelium, and other glial cells, Müller cells constitute the neurovascular unit in the retina that controls the transportation of ions, water, lipids, and proteins inside the BRB. Therefore, Müller cells are critical to maintaining the function and integrity of the retina.

Studies have found that Müller cells are morphologically aberrant or even absent in DR rats. However, the underlying mechanism is still largely unknown. Reduced mitochondrial oxidative phosphorylation has been found to be associated with functionally abnormal in Müller cells, and it results in neuronal dysfunction and retinal structure disruption [5]. Ca^2+^ overload is among the most common causes of mitochondrial disorders. The endoplasmic reticulum (ER) is an important organelle in the cell responsible for protein synthesis, transport, and balance regulation of Ca^2+^. The ER and mitochondria form physical contact sites termed mitochondria-associated ER membranes (MAMs). MAMs play an important role in maintaining calcium homeostasis [6]. ERS upregulates MAM formation and promotes Ca^2+^ efflux from ER into mitochondria. Mitochondrial Ca^2+^ overload subsequently provokes mitochondria permeability transition pore (mPTP) opening and mitochondrial content release. Under normal conditions, DNA is predominantly distributed in nuclei and mitochondria. However, the persistent opening of mPTP leads to the release of mitochondrial DNA (mtDNA), which binds to cGAS in the cytosol and activates STING. As a result, interferon regulatory factor 3 (IRF3) and TANK-binding kinase 1 (TBK1) are phosphorylated, which are required for the production of interferon (IFN) and other inflammatory cytokines. Thus, maintaining mitochondrial function is essential to protect Müller cells and alleviate DR.

Bile acids are cholesterol-derived amphiphilic steroid acids produced in mammals and other vertebrates. Recent studies have demonstrated that bile acids are important signaling molecules that participate in metabolism [7]. Two major types of bile acid receptors have been reported, namely, farnesoid X receptor (FXR) and Takeda G-protein-coupled receptor 5 (TGR5). TGR5 is expressed in retinal microvascular endothelial cells [8]. Recently, we found that TGR5 was also expressed in Müller cells. However, TGR5’s potential role in controlling Müller cell function is poorly understood. We hypothesize that since TGR5 is closely associated with energy metabolism, it may also be relevant to mitochondrial function. The function and regulatory machinery of TGR5 in DR deserve to be explored intensively.

Herein, we demonstrate that TGR5 is a key factor in maintaining mitochondrial function in Müller cells. TGR5 agonist downregulates MAMs formation, which alleviates mitochondrial Ca^2+^ overload-induced mitochondrial dysfunction. As a result, mtDNA release from mitochondria is decreased and inflammatory responses mediated by cGAS-STING signaling is repressed in DR. Collectively, these studies indicate that TGR5 is a promising therapeutic target for DR.

## Materials and methods

### Cell culture and transfection

Human Müller cells were obtained from Bluefbio Biology Technology Development Co., Ltd (Shanghai, China). Müller cells were cultured at 37 °C and 5% CO_2_ in DMEM/F12 medium (Gibco, USA) with 10% FBS (Gibco, USA), 1% penicillin and streptomycin (Beyotime, China).

After reaching 60% confluency, cells were transfected with TGR5 siRNA, GRP75 siRNA or nontarget control siRNA (NC) using riboFECT CP Transfection Kit (RiboBio, China). Briefly, cells were grown overnight in 6-well plates. 120 μL of Opti-MEM medium were mixed with 12 μL of riboFECT CP and 100 nM siRNA. The cells were then harvested 48 h after transfection and transfection efficiency was assessed by western blot. Detailed siRNA information is listed in electronic supplementary material Table S1.

### Animals and streptozotocin (STZ)-induced diabetic rats

Male Sprague-Dawley rats (6–8 weeks, 200–220 g) were purchased from Changzhou Cavans Experimental Animal Co., Ltd (Changzhou, China) and housed in standard conditions (24 ± 2°C, 45 ± 5% humidity, and 12 h light/dark cycle), permitted adequate provision of food and water. All animal experiments were performed according to the Guide for the Care and Use of Laboratory Animals of the National Institutes of Health, and the procedures were approved by the Animal Care and Use Committee of Nanjing Medical University.

STZ-induced diabetic rats: STZ was dissolved in 10 mM citrate buffer (pH 4.5) and given by intraperitoneal injection (i.p.,1.5%, 60 mg/kg) to induce diabetes. The control rats were given the same volume of citrate buffer. Rats with a blood glucose level >16.7 mM for two consecutive measurements were considered diabetic and used in this study.

### TGR5 knockdown and intravitreal injection

An adeno-associated virus serotype 8 (AAV8), allowing for RNAi against TGR5 (AAV8-shTGR5), was constructed and packaged by Vigene Biosciences, Inc. Detailed AAV8-shTGR5 information is presented in Table S1. Rats were randomly grouped into six groups based on body weight. The AAV8-vector group was intravitreally injected with 2 μL AAV8-vector, while the AAV8-shTGR5 and AAV8-shTGR5+H-151 (STING inhibitor) groups were injected with AAV8-shTGR5 (6.25 × 10^12^ viral genomes/mL) with a 33-gauge needle.

Two weeks after STZ injection, rats were anesthetized with an intraperitoneal injection of sodium pentobarbital (50 mg/kg). INT-777 (100 ng/μL, 2 μL), H-151(50 ng/μL, 2 μL), or the same volume of solvent was injected into the vitreous cavity using a 33-gauge needle as previously described [9]. Then, the drugs were administered intravitreally every four weeks for 12 weeks.

### Cell viability

Cells were seeded in 96-well plates (100 μL,1.0 х 10^3^ cells/well) and cell viability was examined by Cell Counting Kit-8 (Beyotime, China). Upon completion of treatment, the culture medium of 96-well plates was removed and 100 μL of CCK-8 solution was added. After incubation with CCK8 for 2–4 h, the absorbance was measured at the wavelength of 450 nm.

### Analysis of mitochondrial morphology

Cells were incubated with Mito Tracker Red (200 nM, Beyotime, China) for 30 min. Then the mitochondrial morphology was observed by confocal microscopy (63×, Leica, Germany) and the mitochondrial length was quantified with ImageJ software as described previously [9].

### Analysis of mitochondrial ROS

To evaluate mitochondrial ROS, cells were incubated with MitoSOX™ Red (5 μM, Thermo Fisher, USA) for 10 min and then incubated with MitoTracker-Green (200 nM, Beyotime, China) for 30 min. The fluorescence was observed by confocal microscopy (40×, Leica, Germany).

### Analysis of mitochondrial Ca^2+^

To measure mitochondrial Ca^2+^, cells were incubated with a mixture of Rhod-2 (2 μM, YEASEN, China) and Pluronic F-127 (0.02%, Beyotime, China) for 30 min. Next, the cells were incubated with MitoTracker Green (200 nM, Beyotime, China) for 30 min. The fluorescence was observed using a confocal microscope (40×, Leica, Germany).

### Measurement of mitochondrial membrane potential

The mitochondrial membrane potential (MMP) was detected by a JC-1 staining kit (Beyotime, China). The ratio of red (aggregates) to green (monomers) fluorescence represents the changes in MMP. When the ratio decreases, mitochondria depolarize and serve as an early indicator of apoptosis. Cells were washed thrice with the buffer, after which they were incubated with the JC-1 working solution for 20 min in accordance with the manufacturer’s instructions. The fluorescence was observed using a confocal laser scanning microscope (40×, Leica, Germany), and the ratio of aggregates/monomers was calculated using the ImageJ software.

### Determination of mitochondrial permeability transition pore

Mitochondrial permeability transition pore (mPTP) opening was monitored using the mPTP assay kit (Beyotime, China). Calcein AM is a fluorescent dye for living cells that accumulates in the mitochondria and excites green fluorescence. When it forms a complex with Co^2+^, the fluorescence is quenched. The mixtures of Calcein AM (2 μM, Beyotime, China) and CoCl_2_ (250 μM) were added to the cells incubating for 30 min, then cultured in DMEM/F12 supplemented with 10% FBS for 40 min. After that, cells were incubated with MitoTracker Red (200 nM, Beyotime, China) for 30 min. Finally, the cells were observed with a laser scanning confocal microscope (40×, Leica, Germany).

### Enzyme-linked immunosorbent assay

The levels of 2’3’-cGAMP in cells were determined with commercial enzyme-linked immunosorbent assay (ELISA) kits (Cayman, USA) according to the manufacturer’s instructions.

### Detection of ER-mitochondria interactions

To detect ER-mitochondria interactions, cells were incubated with ER-Tracker Green (1 μM, Beyotime, China) for 20 min, followed by MitoTracker Red (200 nM, Beyotime, China) for 30 min. After being washed thrice, the fluorescence was observed under a laser scanning confocal microscope (40×, Leica, Germany).

### Western blotting

Cells or retina tissues were lysed on ice with lysis buffer and centrifuged at 12,000 *g* at 4 °C for 15 min to get the supernatant, then the supernatant was added to 5× SDS-PAGE loading buffer and the protein lysates were boiled at 95 °C for 5 min. Protein concentration was determined by BCA Kit (Beyotime, China). Protein samples were separated by SDS polyacrylamide gel electrophoresis and then transferred to PVDF membranes. After that, the membranes were blocked in 5% milk for 2 h and then incubated with primary antibodies: p-DRP1 (1:1000, #4494, CST), DRP1 (1:1000, ab184247, Abcam), cGAS (1:1000, #15102, CST), p-STING (1:1000, #50907, CST), STING (1:1000, #13647, CST), p-IRF3 (1:1000, #29047, CST), IRF3 (1:1000, #11904, CST), p-NF-κB (1:1000, #3033, CST), NF-κB (1:1000, #8242, CST), IL-6 (1:1000, #12153, CST), TNF-α (1:1000, #12153, CST), IFN-β (1:1000, #6945, CST), IP3R1 (1:200, sc-271197, Santa), GRP75 (1:1000, #3593, CST), VDAC1 (1:200, sc-390996, Santa) and β-actin (1:5000, 66009-1-Ig, Proteintech) were incubated at 4 °C overnight. After incubation with HRP-linked antibodies, protein bands were visualized with chemiluminescence kit (Thermo Fisher Scientific, USA).

### Co-immunoprecipitation assays

The procedure was performed following the manufacturers’ instructions with minor modifications [10]. Briefly, after reaching 95% confluency, cells were lysed on ice for 30 min with Pierce™ IP lysis buffer (Thermo Fisher, USA) containing protease inhibitor (Roche, Switzerland). Then the lysates were centrifuged at 12,000 g for 15 min at 4 °C to get the supernatant. After that, the supernatant was incubated with 2 μg primary antibodies IP3R1 (sc-271197, Santa), VDAC1 (sc-390996, Santa), or IgG (sc-2025, Santa) at 4 °C overnight. Subsequently, the lysates were incubated with 50 μl protein A/G magnetic beads (MCE, USA) at 4 °C for 3 h, and beads were then washed four times with the IP buffer. Finally, protein A/G magnetic beads were eluted by boiling in 1×SDS sample buffer before western blot analysis.

### MtDNA extraction and RT-PCR

MtDNA was extracted using a DNA Extraction Kit (Qiagen) after mitochondrial isolation. Briefly, cells were digested with trypsin. A mitochondrial isolation reagent with PMSF (Beyotime, China) was added in cells, and then cells were homogenized. The homogenate was centrifuged three times to pellet the mitochondria according to the manufacturers’ protocols. The pellet was washed once with homogenization buffer and centrifuged for 5 min at 9000 *g*. The mitochondrial pellet was resuspended in PBS, and mtDNA was extracted.

RT-PCR was performed using the Fast SYBR Green Master Mix (Roche, Switzerland). The detailed primer sequences are listed in Table S2.

### Immunofluorescence (IF)

Cells or frozen sections were fixed with 4% formaldehyde, permeabilized with 0.2% Triton-100 for 30 min, and blocked with 5% BSA for 2 h at room temperature. The samples were then incubated with primary antibodies TGR5 (1:50, ab72608, Abcam), Glutamine synthetase (GS, 1:50, ab64613, Abcam), Tom 20 (1:250, ab186735, Abcam), dsDNA (1:100, sc-58749, Santa), cGAS (1:100, #15102, CST), IP3R1 (1:100, sc-271197, Santa), GRP75 (1:100, #3593, CST), VDAC1 (1:100, sc-390996, Santa), or Glial fibrillary acidic protein (GFAP, 1:200, G3893, Sigma-Aldrich) at 4 °C overnight. After washing, the samples were incubated with fluorochrome-conjugated secondary antibody (1:400, Thermo Fisher) at room temperature for 2 h, and then incubated with DAPI for 10 min. Finally, the samples were observed by a confocal microscope (Leica, Germany).

### Immunohistochemistry (IHC)

Paraffin sections (4 μm) were dewaxed in xylene and rehydrated with graded ethanol. Microwave antigen retrieval was carried out in 10 mM citrate buffer and then the sections were treated with 3% H_2_O_2_ for 15 min to eliminate endogenous peroxidase activity and blocked with 5% BSA for 1 h. Next, samples were incubated with VEGF (1:200, sc-7269, Santa), 8-OHdG (1:100, sc-393871, Santa) overnight at 4 °C and then incubated with secondary antibodies. DAB incubated and stained with Mayer’s hematoxylin, finally, sections were visualized under a light microscope (Olympus, Japan).

### Retinal imaging

Rats were anesthetized, and then pupils were dilated with Cyclomydril (Alcon, Fort Worth, TX, USA). Optical coherence tomography (OCT) was performed using Phoenix Micron IV (Phoenix Research labs, California) retinal imaging microscope according to the manufacturers’ instruction.

### Retinal vascular permeability assay

BRB integrity was assessed using Evans Blue. Briefly, Evans blue dye (3%) was injected into the tail vein. About 2 h later, rats were exsanguinated and perfused with ice-cold saline. Then retinas were carefully peeled and mounted on a glass slide. Extravasation of Evans blue dye was examined under an Olympus BX-51 light microscope (Olympus, Japan).

### Periodic acid-schiff (PAS) staining

Periodic acid-Schiff (PAS) staining was used to evaluate retinal vasculature changes. Eyes were enucleated and fixed in 4% paraformaldehyde for 2 h and then incubated with 3% trypsin at 37 °C for 2 h. The retinas were gently shaken to get free vessel network. Then, the vessel network was dried on glass slides for 2 h and was stained with PAS kit (Solarbio, China). The acellular capillaries were examined using light microscopy (Olympus, Japan).

### Transmission electron microscopy imaging

The eye samples were fixed in 2.5% glutaraldehyde for 2 h at room temperature, pruned into 2 × 3 mm pieces, and then, the protocol described previously was followed [11]. The ultrastructure of the retinal tissues was observed under a transmission electron microscope (JEOL JEM-1400Flash, Japan).

### Hematoxylin and eosin (H&E) staining

Eyes were enucleated and fixed in 4% paraformaldehyde for 48 h, and then dehydrated in a graded series of ethanol and embedded in paraffin. Paraffin sections (4 μm) of retinas were subjected to hematoxylin and eosin (H&E) staining according to the manufacturer’s instructions (BOSTER, China).

### Statistical analysis

All results were expressed as mean ± SD in three independent experiments. Datasets were analyzed using the Student’s *t*-test or single-factor ANOVA with Tukey’s post-hoc analysis. The sample size was predetermined based on published literatures and previous lab experience. No statistical methods were used to predetermine the sample size. Rats were randomly allocated to experimental groups. Investigators were not blinded to the animal intravitreal injection. No samples were excluded from the analysis. Normal distribution of data was verified by a Shapiro–Wilkinson test. The variance was similar between the groups that were being statistically compared. Data analyses were performed using The GraphPad Prism version 7.00 (GraphPad Software, San Diego, CA), and *P*-values < 0.05 were considered statistically significant.

## Results

### TGR5 activation alleviates Müller cell injury by reducing mitochondrial damage in DR

**Fig. 1:**
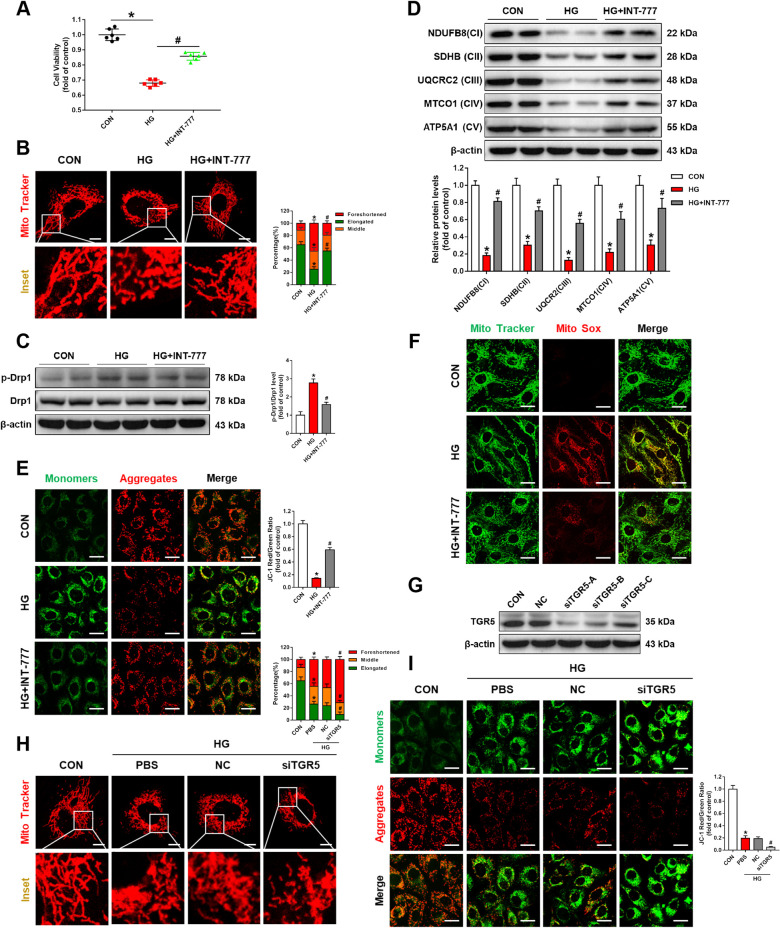
TGR5 activation alleviates Müller cell injury by reducing mitochondrial damage in DR. Müller cells were treated with HG (high glucose, 33 mM) for 48 h in DMEM with 2% FBS in the presence or absence of INT-777 (**A–F**) or TGR5 siRNA (**G–I**). The INT-777 (30 μM) were added 2 h before HG stimulation, and also existed during HG stimulation. For siRNA assay, transfection was done 24 h before HG treatment. **A** Cell proliferation was determined by CCK-8 (*n* = 6). **B** Representative confocal images and bar graph showing mitochondrial network morphology. Scale bars = 10 μm. **C** Representative western blot showing p-Drp1and Drp1 protein levels (*n* = 3). **D** Representative western blot showing NDUFB8, SDHB, UQCRC2, MTCO1, and ATP5A1 protein levels in Müller cells (*n* = 3). **E** Representative confocal images of JC-1 staining and bar graph showing MMP. Scale bars = 25 μm. **F** Representative confocal images of MitoSOX™ Red and Mito-Tracker Green staining showing mtROS in Müller cells. Scale bars = 25 μm. **G** Western blot shows transfection efficiency of TGR5 in Müller cells. **H** Representative confocal images and bar graph showing mitochondrial network morphology in Müller cells transfected with TGR5 siRNA under HG stimulation. Scale bars = 10 μm. **I** Representative confocal images of JC-1 staining and bar graph showing MMP in Müller cells. Scale bars = 25 μm. Data are presented as mean ± SD, ^*^p < 0.05 versus control (CON) group, ^#^*p* < 0.05 versus HG group.

Müller glial cells extend throughout the retina and function to maintain retinal homeostasis and integrity [12]. Immunofluorescence staining revealed co-localization of TGR5 and glutamine synthetase (GS, a marker of Müller cells) in the frozen retinal sections, implying that TGR5 is expressed in Müller cells (Fig. S1). Given that Müller cells are key players in DR progression, we first investigated the effect of TGR5 on Müller cell viability under high glucose (HG) condition. We found that INT-777, a TGR5 agonist, remarkably rescued HG-induced decrease in cell viability (Fig. 1A).

Next, we wanted to determine the mechanism by which activation of TGR5 attenuated Müller cell injury. TGR5 is involved in the regulation of energy metabolism [13]. Mitochondrial function has major impacts on cellular energy metabolism, and we hypothesize that TGR5 may also be relevant to mitochondrial function. Given that mitochondrial function is closely associated with its morphology, we examined mitochondrial morphology in Müller cells using MitoTracker Red, a red-fluorescent dye that stains mitochondria in live cells. We found that mitochondria in HG-treated cells were fragmented into short spheres, whereas INT-777 treatment significantly restored mitochondrial network morphology (Fig. 1B). To exclude impacts of osmolarity difference, the cells were treated with L-Glucose (33 mM) or Mannitol (33 mM) under the same conditions. We found that neither L-Glucose nor Mannitol influenced cell viability and mitochondrial network (Fig. S2A, B). Drp1 is a crucial executor of mitochondrial fission, and phosphorylation of DRP1 at Ser616 is a marker of Drp1 activation [14]. Western blot indicated that the TGR5 agonist inhibited p-Drp1(Ser616) expression (Fig. 1C). Drp1 was able to be phosphorylated by PKCδ [15]. Notably, INT-777 inhibited HG-induced PKCδ phosphorylation (Fig. S3A). Furthermore, TGR5 knockdown exacerbated HG-induced phosphorylation of Drp1 and PKCδ (Fig. S3B, C). Meanwhile, PKCδ inhibitors reversed siTGR5-induced Drp1 phosphorylation under HG stimulation (Fig. S3B, C). The above results showed that TGR5 might regulate mitochondrial fission through the PKCδ-Drp1 pathway.

**Fig. 2:**
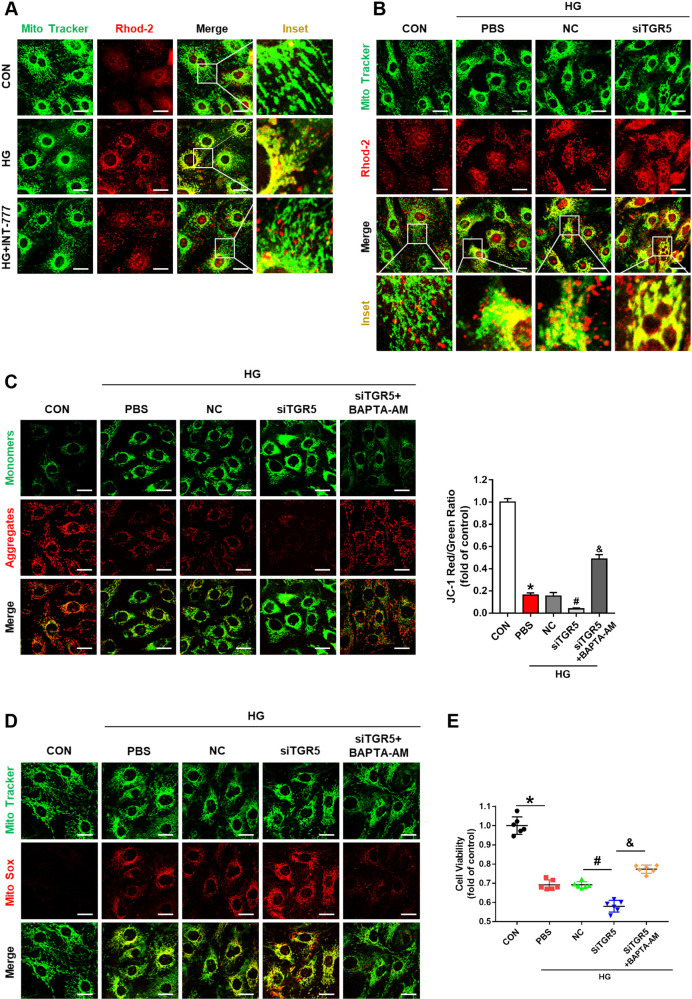
TGR5 regulates mitochondrial function by modulating mitochondrial Ca^2+^. **A** Representative confocal images of Rhod-2 Red and MitoTracker Green staining showing mitochondrial Ca^2+^ in Müller cells under HG conditions with or without INT-777. Scale bars = 25 μm. **B** Representative confocal images of Rhod-2 red and MitoTracker Green staining showing mitochondrial Ca^2+^ in Müller cells transfected with TGR5 siRNA. Scale bars = 25 μm. **C** Representative confocal images of JC-1 staining and bar graph showing MMP in Müller cells transfected with control siRNA (NC) and those transfected with TGR5 siRNA in the absence or presence of BAPTA-AM. Scale bars = 25 μm. **D** Representative confocal images of MitoSOX™ Red and MitoTracker Green staining showing mtROS in Müller cells transfected with control siRNA (NC) and those transfected with TGR5 siRNA in the absence or presence of BAPTA-AM. Scale bars = 25 μm. **E** Cell viability was determined by CCK-8 (*n* = 6). Data are expressed as mean ± SD, ^*^p < 0.05 versus control group (CON), ^#^*p* < 0.05 versus HG group. ^&^*p* < 0.05 versus TGR5 knockdown (siTGR5) group.

Mitochondria are the principal sites of energy conversion where ATP is generated through the process of oxidative phosphorylation (OXPHOS) [16]. The expressions of mitochondrial respiratory chain complexes such as NDUFB8, SDHB, UQCRC2, MTCO1, and ATP5A1 were upregulated by INT-777 (Fig. 1D), suggesting that TGR5 activation improved mitochondrial function. MMP is an indicator of mitochondrial state [17]. JC-1 staining of the mitochondria showed that MMP decreased with HG stimulation but was significantly alleviated by INT-777 (Fig. 1E). The level of mitochondrial ROS (mtROS) was measured by MitoSOX staining. As depicted in Fig. 2F, HG-treated cells displayed significantly increased MitoSOX fluorescence, whereas INT-777 treatment showed reduced MitoSOX staining.

To further explore the role of TGR5 in mitochondrial dysfunction, Müller cells were transfected with TGR5-specific siRNA (Fig. 1G). TGR5 knockdown aggravated HG-induced mitochondrial fragmentation (Fig. 1H). Besides, downregulation of TGR5 led to a decrease in MMP (Fig. 1I). Thus, we conclude that TGR5 acts as a key factor in mediating mitochondrial function in Müller cells.

### TGR5 regulates mitochondrial function by modulating mitochondrial Ca^2+^

Mitochondrial Ca^2+^ homeostasis is closely associated with mitochondrial function [18]. We next examined Ca^2+^ content in the mitochondria by loading Müller cells with the mitochondrial calcium probe Rhod-2 [19]. Representative images of co-localized mitochondrial tracker and Rhod-2 are presented in Fig. 2A. HG treatment caused a significant increase in the Rhod-2 signal, indicating Ca^2+^ accumulation in the mitochondria. On the other hand, mitochondrial Ca^2+^ decreased significantly following treatment with INT-777. Mitochondrial Ca^2+^ was further increased after TGR5 knockdown (Fig. 2B). Furthermore, TGR5 knockdown aggravated MMP depolarization and increased mtROS (Fig. 2C, D). To gain further insight into the role of Ca^2+^ in TGR5 signaling, Ca^2+^ chelator BAPTA-AM (5 μM) was given after TGR5 knockdown, and MMP and mtROS were detected. We noted that BAPTA-AM reversed TGR5 silencing-induced impairments (Fig. 2C, D). In addition, the CCK-8 assay further demonstrated that BAPTA-AM could improve cell viability (Fig. 2E). These results suggested that TGR5 regulates mitochondrial function by modulating mitochondrial Ca^2+^.

### TGR5 affects mitochondrial Ca^2+^ homeostasis through regulating MAMs

**Fig. 3:**
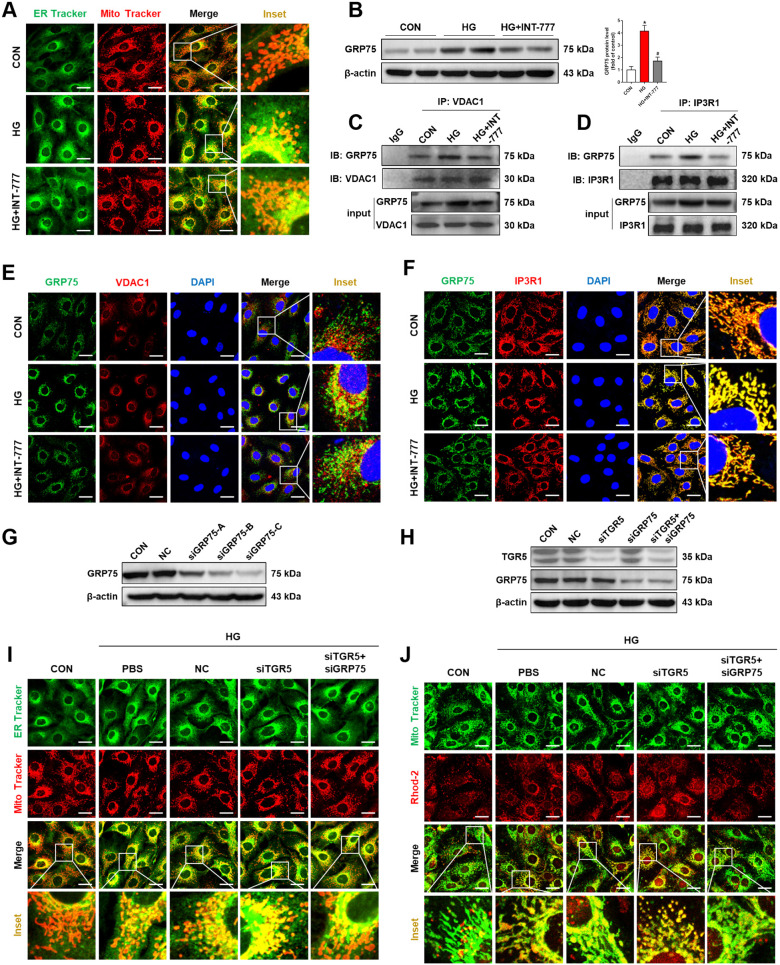
TGR5 affects mitochondrial Ca^2+^ homeostasis by regulating MAM. Müller cells were treated with HG in the presence or absence of INT-777 (**A–F)** or transfected with TGR5 siRNA and treated with or without GRP75 siRNA under HG stimulation (**G–I**). **A** Representative confocal images showing co-localization of ER (green) and mitochondria (red) in Müller cells. Scale bars = 25 μm. **B** Representative western blot showing GRP75 protein level (*n* = 3). **C** Immunoprecipitation showing the interaction between GRP75 and VDAC1 in Müller cells. **D** Immunoprecipitation showing the interaction between GRP75 and IP3R1 in Müller cells. **E** Representative immunofluorescence images showing co-localization of GRP75 (green) and VDAC1 (red). Blue=DAPI; yellow=merge. Scale bar = 25 μm. **F** Representative immunofluorescence images showing co-localization of GRP75 (green) and IP3R1 (red). Blue=DAPI; yellow=merge. Scale bar = 25 μm. **G** Representative western blot showing the transfection efficiency of GRP75 in Müller cells. **H** Representative western blot showing the transfection efficiency of GRP75/TGR5 double knockdown in Müller cells. **I** Representative confocal images of MitoSOX™ Red and MitoTracker Green staining showing mtROS in Müller cells transfected with TGR5 siRNA and treated with or without GRP75 siRNA. Scale bar = 25 μm. **J** Representative confocal images of Rhod-2 Red and MitoTracker Green staining showing mitochondrial Ca^2+^ in Müller cells. Scale bar = 25 μm. Data are expressed as mean ± SD, *p < 0.05 versus CON group, ^#^*p* < 0.05 versus HG group. ^&^*p* < 0.05 versus siTGR5 group.

ER is the site of Ca^2+^ storage [20]. TGR5 inhibits HG-induced increase in mitochondrial Ca^2+^ (Fig. 2A). As stated earlier, ER and mitochondria form physical contact sites, MAMs, which mediate the Ca^2+^ efflux from ER to mitochondria [21]. Co-localization of MitoTracker and ER-Tracker is increased in HG-treated cells, suggesting an increase in MAM formation. After INT-777 treatment, co-localization between mitochondria and ER was remarkably reduced (Fig. 3A). Thus, we speculated that TGR5-mediated mitochondrial Ca^2+^ homeostasis was regulated by MAMs. The IP3R1-GRP75-VDAC1 axis is shown to be present at the MAMs and is responsible for Ca^2+^ transportation from ER to mitochondria [22]. GRP75 showed a significant upregulation under the HG condition, while TGR5 activation reversed the elevation (Fig. 3B).

There are predominantly two types of intracellular calcium release channels, IP3R and ryanodine receptors (RYR). We found that RYR was upregulated by HG treatment but INT-777 does not reverse the alteration (Fig. S4). We next sought to ascertain whether the IP3R1-GRP75-VDAC1 axis would change upon HG and TGR5 agonist treatment. Co-immunoprecipitation and immunofluorescence in Müller cells revealed that HG incubation increased the binding between GRP75 and VDAC1 (Fig. 3C, E) as well as the binding between GRP75 and IP3R1 (Fig. 3D, F). These binding capacities were inhibited by INT-777 treatment. To further examine the role of GRP75 in TGR5-mediated mitochondrial Ca^2+^ homeostasis, Müller cells were transfected with a control siRNA, a TGR5-specific siRNA, or co-transfected with a TGR5- and GRP75-specific siRNA. Knockdown efficiency is depicted in Fig. 3G, H. Compared with the HG group, TGR5 knockdown promoted MAM formation of and decreased mitochondrial Ca^2+^ levels (Fig. 3I, J). However, the effects of TGR5 silencing on MAM formation and mitochondrial Ca^2+^ overload were significantly reversed by GRP75 silencing (Fig. 3I, J). In addition, GRP75 shRNA could ameliorate AAV-shTGR5-induced aggravation of retinal dysfunction in DR rats (unpublish data). These findings confirmed that suppression of GRP75 expression by TGR5 led to a block of IP3R1-GRP75-VDAC1 complex formation, which in turn decreased mitochondrial Ca^2+^ levels.

### Activation of TGR5 maintains mitochondrial homeostasis and inhibits cGAS-STING-mediated inflammatory response

**Fig. 4:**
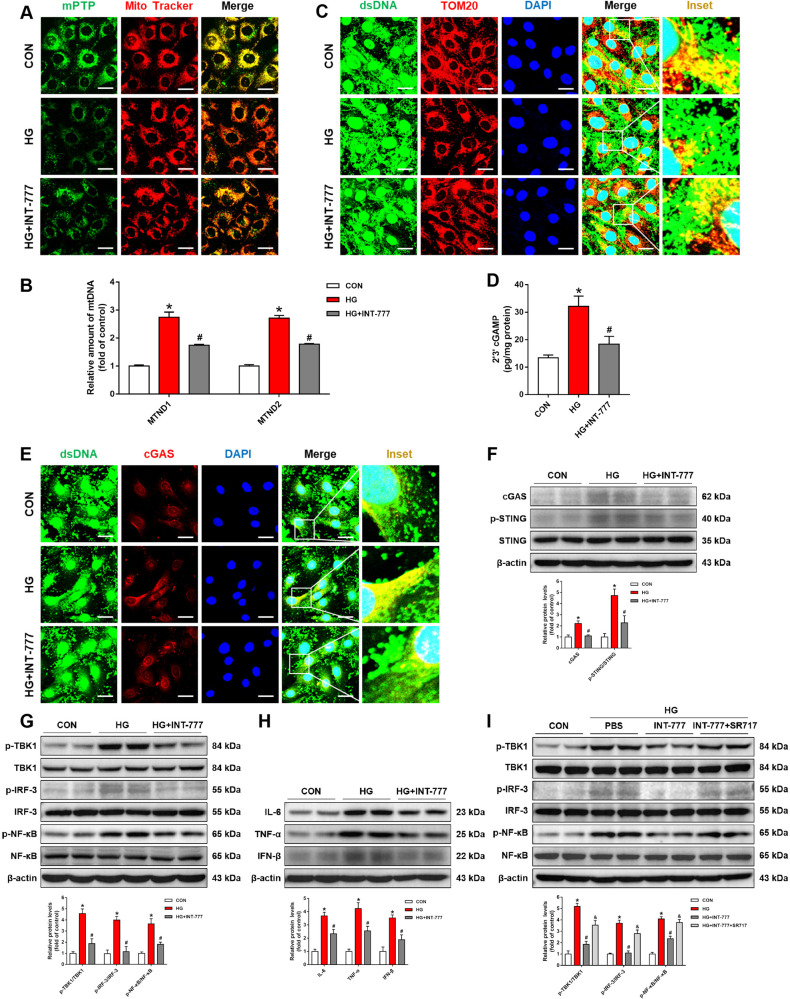
Activation of TGR5 maintains mitochondrial homeostasis and inhibits cGAS-STING-mediated inflammatory response. Müller cells were treated with HG in the presence or absence of INT-777. **A** Representative confocal images showing mPTP opening. Scale bars = 25 μm. **B** qRT-PCR assay showing mRNA levels of MTND1 and MTND-2 (*n* = 3). **C** Representative immunofluorescence images showing co-localization of dsDNA (green) and Tom20 (red). Blue=DAPI; yellow=merge. Scale bars = 25 μm. **D** ELISA assay showing the level of 2’3’-cGAMP in Müller cells (*n* = 3). **E** Representative immunofluorescence images showing co-localization of dsDNA (green) and cGAS (red). Blue=DAPI; yellow=merge. Scale bars = 25 μm. **F** Representative western blot showing cGAS, p-STING, and STING levels in Müller cells (*n* = 3). **G** Representative western blot showing p-TBK1, TBK1, p-IRF3, IRF3, p-NF-κB, and NF-κB levels in Müller cells (*n* = 3). **H** Representative western blot showing IL-6, TNF-α, and IFN-β levels (*n* = 3). **I** Representative western blot showing p-TBK1, TBK1, p-IRF3, IRF3, p-NF-κB, and NF-κB levels in Müller cells with or without SR717 (*n* = 3). Data are expressed as mean ± SD, *p < 0.05 versus CON group, ^#^*p* < 0.05 versus HG group.

High, sustained levels of mitochondrial Ca^2+^ resulted in mPTP opening and the release of mitochondrial contents into the cytosol [23]. The previous results suggested a distinct role of INT-777 in blocking mitochondrial Ca^2+^ overload. In this study, we found that the TGR5 agonist reversed HG-induced mPTP opening (Fig. 4A). NADH dehydrogenase-(MTND)1 and MTND2 are the crucial signatures of mtDNA [24]. To determine whether HG induced the release of mtDNA from the mitochondria into the cytosol, we collected cells and fractionated them to separate cytosolic (cyto) and mitochondrial (mito) fractions and then performed quantitative real-time PCR (qRT-PCR). Our results suggested that HG markedly increased cytosolic levels of MTND1 and MTND2, while INT-777 treatment blocked the increase in cytosolic mtDNA level (Fig. 4B). To further confirm whether TGR5 inhibited mtDNA release from mitochondria, we stained the cells with both dsDNA and TOM20 for co-localization imaging. Confocal images revealed the co-localization between DNA and mitochondria in control cells and a decreased co-localization in HG-treated cells. INT-777 treatment could reduce the level of cytoplasmic DNA (Fig. 4C).

Cytoplasmic mtDNA binds to cGAS and leads to the production of 2’3’ cyclic GMP-AMP (cGAMP). We noted that INT-777 treatment rescued HG-induced co-localization between DNA and cGAS in cells (Fig. 4D). INT-777 decreased the intracellular content of cGAMP, further corroborating the inhibitory effect of TGR5 on cGAS activity (Fig. 4E). Meanwhile, INT-777 inhibited the expression of cGAS (Fig. 4F). cGAMP is a second messenger as well as a potent agonist of STING. HG-induced phosphorylation of STING, whereas INT-777 reversed the aberrant activation (Fig. 4F). STING activation triggered recruitment and phosphorylation of TBK1, which further phosphorylated IRF3 and NF-κB. Interestingly, INT-777 also suppressed STING-mediated phosphorylations of TBK1, IRF3, and NF-κB (Fig. 4G). Nuclear transcription factors such as IRF3 and NF-κB mediated the expressions of inflammatory factors, namely, TNF-α, interleukin-6 (IL-6), and IFN-β. In accordance with our previous results, the TGR5 agonist reversed HG-induced upregulation of TNF-α, IL-6, and IFN-β (Fig. 4H). To further verify the role of cGAS-STING signaling in TGR5-mediated inflammatory response, SR717 (STING agonist, 10 μM) was given prior to the treatment of INT-777. We found that INT-777 suppressed the phosphorylations of TBK1, IRF3 and NF-κB, while SR717 blocked this phenomenon (Fig. 4I). Collectively, these results indicated that TGR5 maintained mitochondrial homeostasis and inhibited cGAS-STING signal-mediated inflammatory response in DR.

### TGR5 knockdown disrupts mitochondrial homeostasis and promotes cGAS-STING-mediated inflammatory response

**Fig. 5:**
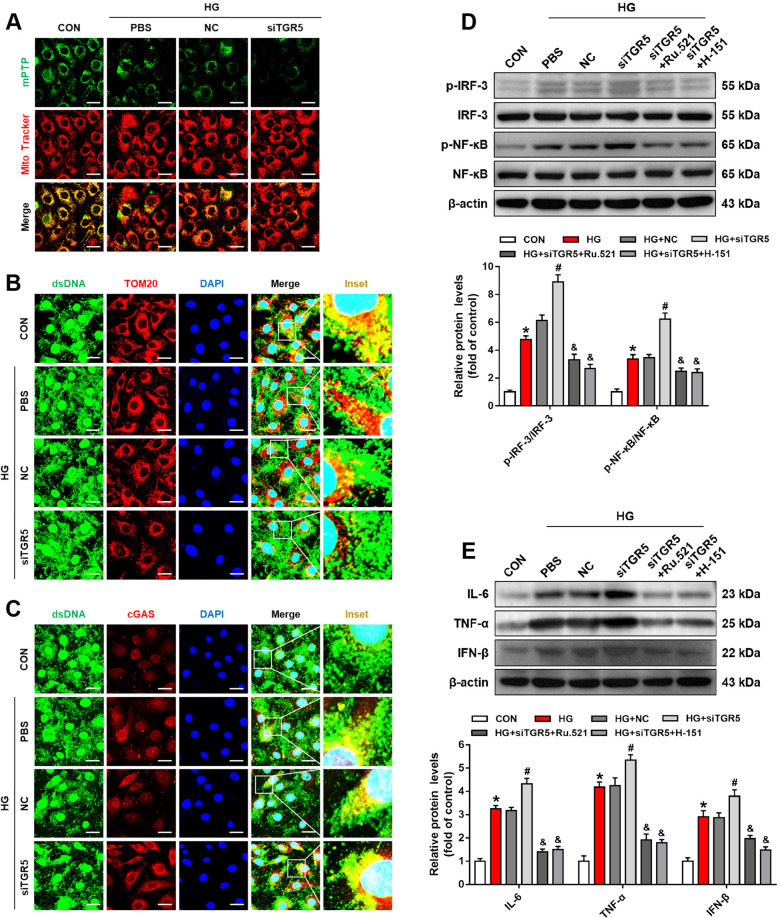
TGR5 knockdown disrupts mitochondrial homeostasis and promotes cGAS-STING-mediated inflammatory response. Müller cells were transfected with TGR5 siRNA under HG stimulation (**A–C**). **A** Representative confocal images showing mPTP opening in Müller cells. Scale bars = 25 μm. **B** Representative immunofluorescence images showing co-localization of dsDNA (green) and Tom20 (red). Blue=DAPI; yellow=merge. Scale bars = 25 μm. **C** Representative immunofluorescence images showing co-localization of dsDNA (green) and cGAS (red). Blue=DAPI; yellow=merge. Scale bars = 25 μm. Müller cells were transfected with TGR5 siRNA and treated with or without cGAS/STING inhibitors under HG stimulation (**D, E**). **D** Representative western blot showing p-IRF3, IRF3, p-NF-κB, and NF-κB protein levels in Müller cells (*n* = 3). **E** Representative western Blot showing IL-6, TNF-α, and IFN-β protein levels in Müller cells (*n* = 3). Data are shown as mean ± SD, *p < 0.05 versus CON group, ^#^*p* < 0.05 versus HG group. ^&^*p* < 0.05 versus siTGR5 group.

To further clarify the regulatory role of TGR5 on mitochondrial homeostasis and the cGAS-STING pathway, TGR5 knockdown was performed in the following experiments. As evidenced by our results, TGR5 knockdown accelerated HG-induced mPTP opening (Fig. 5A). Furthermore, TGR5 siRNA decreased the co-localization between DNA and mitochondria under HG stimulation, implying that TGR5 silencing could increase the level of cytoplasmic DNA (Fig. 5B). In addition, TGR5 siRNA promoted HG-induced co-localization between DNA and cGAS (Fig. 5C). To further define the regulatory role of TGR5 on the cGAS-STING pathway, Ru.521 (cGAS inhibitor, 5 μM) or H-151 (STING inhibitor, 5 μM) was used in TGR5 knockdown cells. Phosphorylations of IRF3 and NF-κB were further upregulated in the TGR5 siRNA group compared with HG-treated cells (Fig. 5D). Notably, both cGAS inhibitor and STING inhibitor suppressed phosphorylations of IRF3 and NF-κB in TGR5-knockdown cells (Fig. 5D). Besides, inflammatory factors, namely, TNF-α, IL-6, and IFN-β were increased, and these effects were reversed by cGAS or STING inhibitors (Fig. 5E). To sum up, the above results further confirmed that TGR5 maintained mitochondrial homeostasis and in turn inhibited cGAS-STING signal-mediated inflammatory response.

### TGR5 activation blocks cGAS-STING-mediated inflammatory response and alleviates retinal injury in DR rats

**Fig. 6:**
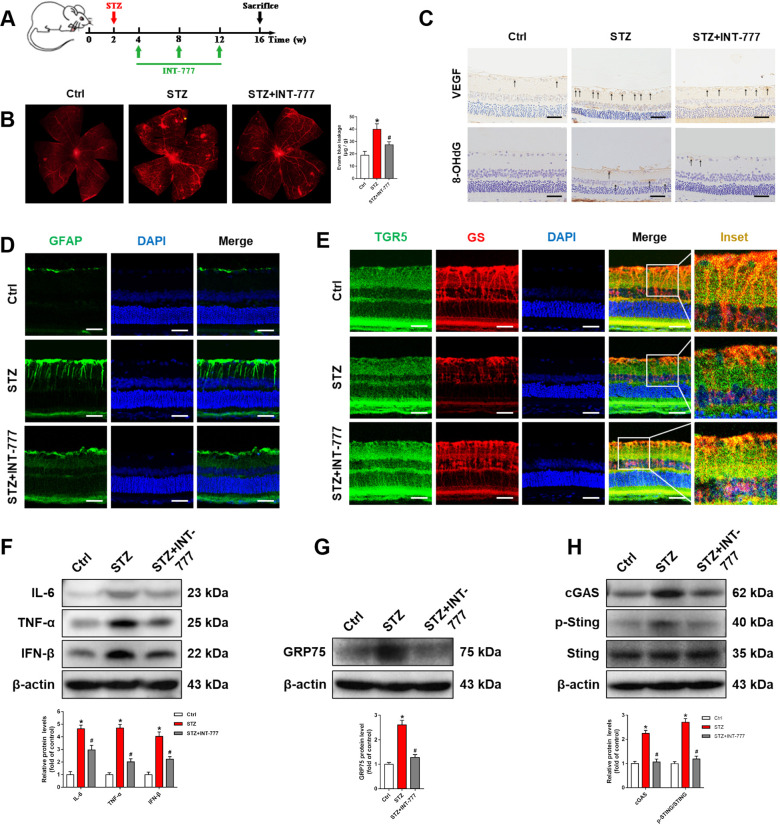
TGR5 activation blocks cGAS-STING-mediated inflammatory response and alleviates retinal injury in DR rats. **A** Flowchart of the in vivo experimental procedures. **B** Representative images of EB staining and bar graph showing retinal vascular leakage in rats (*n* = 3). **C** Representative immunohistochemistry analysis showing VEGF and 8-OHdG expressions in rat retinal sections. Scale bars = 50 μm. **D** Representative immunofluorescence images showing expression of GFAP in rat retinal sections. Blue=DAPI. Scale bars = 25 μm. **E** Representative immunofluorescence images showing co-localization of TGR5 (green) and GS (red) in rat retinal sections. Blue=DAPI; yellow=merge. Scale bars = 25 μm. **F** Representative western blot showing IL-6, TNF-α, and IFN-β protein levels in rat retinas (*n* = 3). **G** Representative western blot showing GRP75 protein level in rat retinas (*n* = 3). **H** Representative western blot showing cGAS, p-STING, and STING protein levels in rat retinas (*n* = 3). Data are shown as mean ± SD, *p < 0.05 versus control (Ctrl) group, ^#^*p* < 0.05 versus diabetic (STZ) group.

To further elucidate the role of TGR5 in DR, STZ-induced DR rats were used in the following experiments. As shown in Fig. 6A, the DR rat model was established by intraperitoneal injection of STZ (60 mg/kg). Two weeks after STZ injection, diabetic rats were administered either INT-777 (100 ng/μL, 2 μL) or vehicle every four weeks for three months. Retinal vascular leakage is a hallmark manifestation of DR. EB staining revealed that vascular leakage was significantly attenuated by INT-777 (Fig. 6B). Müller cells are a rich source of angiogenesis-promoting growth factors, in particular, VEGF. The expression of VEGF is increased in DR rats, while INT-777 inhibited the abnormal elevation (Fig. 6C). 8-Hydroxy-2′-deoxyguanosine (8-OHdG), a marker of oxidative DNA damage, was also increased in DR rats (Fig. 6C). INT-777 inhibited the upregulation of 8-OHdG in DR, indicating that DNA damage in the retina was repaired to a certain extent (Fig. 6C). Glial activation was prominent in the damaged retina, and a high expression of the glial fibrillary acidic protein (GFAP) confirmed that the astrocytes were activated in DR rats. Our results indicated that INT-777 inhibited DR-induced hyperactivation of astrocytes (Fig. 6D). We also observed abnormalities in Müller cell morphology in diabetic rats, and this phenomenon was ameliorated by INT-777 (Fig. 6E). Besides, western blot analysis revealed that INT-777 significantly lowered expressions of inflammatory cytokines TNF-α, IL-6, and IFN-β in the retina (Fig. 6F). Consistent with in vitro assays, GRP75 was upregulated, and cGAS-STING signaling was activated in the retina of DR rats (Fig. 6G, H). However, GRP75, cGAS, and p-STING were downregulated by INT-777 (Fig. 6G, H). In summary, TGR5 activation blocked cGAS-STING-mediated inflammatory response and alleviated retinal injury in DR rats.

### STING inhibitor ameliorates AAV-shTGR5-induced aggravation of retinal dysfunction in DR rats

**Fig. 7:**
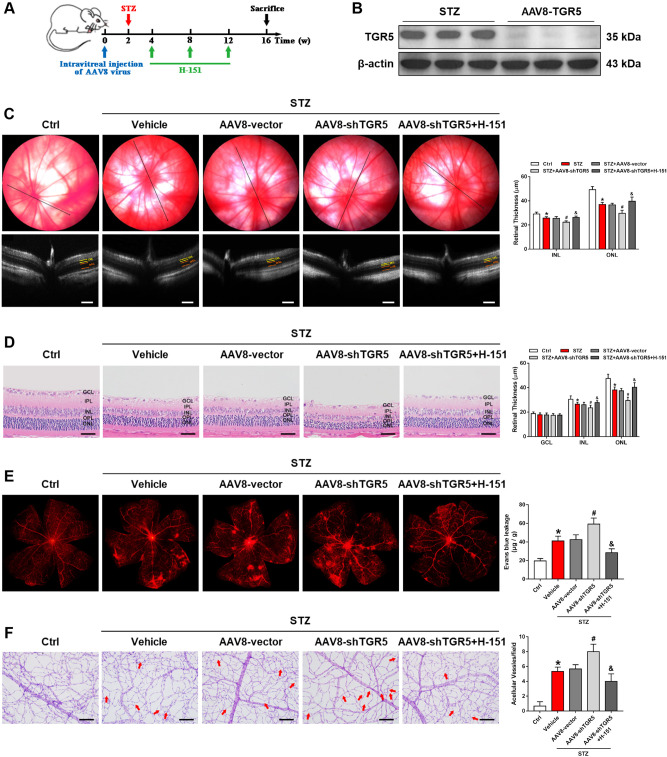
STING inhibitor ameliorates AAV-shTGR5-induced aggravation of retinal dysfunction in DR rats. **A** Flowchart of experimental procedures. **B** Representative western blot showing the efficiency of AAV-mediated TGR5 knockdown in rat retinas. **C** Representative fundus and OCT images showing the thickness of INL and ONL layers in rat retinas. Scale bars = 100 μm. **D** Representative images of H&E staining and bar graph showing the morphological changes in rat retinas. Scale bars = 50 μm. **E** Representative images of EB staining and bar graph showing retinal vascular leakage in rats (*n* = 3). **F** Representative images of PAS staining and bar graph showing retinal acellular capillaries in rat retinas. Scale bars = 50 μm. Data are presented as mean ± SD, *p < 0.05 versus Ctrl group, ^#^*p* < 0.05 versus scrambled AAV8 (AAV8-Vector) group. ^&^*p* < 0.05 versus TGR5 knockdown AAV8 (AAV8-shTGR5) group.

Next, we explored the relationship between TGR5 and cGAS-STING. Adeno-associated virus-shTGR5 (AAV-shTGR5) was used to knock down TGR5 in DR rat retinas. Rats received a single intravitreal injection of AAV-shTGR5 or AAV8-vector in the first week (Fig. 7A). Two weeks later, the DR rat model was established by intraperitoneal injection of STZ (60 mg/kg). Two weeks after STZ injection, the rats were injected with either H-151 (50 ng/μL, 2 μL) or vehicle every four weeks for three months. We first examined the efficiency of AAV-shTGR5 in silencing the expression of TGR5, and the data is illustrated in Fig. 7B. Thinning of the retina can be presented as a sign of retinal degradation. OCT imaging of the retina and H&E staining showed that the outer nuclear layer (ONL) and the inner nuclear layer (INL) of the retina were thinner in AAV-shTGR5 compared with the AAV8-vector group. However, the phenomenon was reversed when H-151 was administered (Fig. 7C, D). Retinal vascular leakage and acellular capillaries are typical indicators of microvascular abnormalities in DR. AAV-shTGR5 exacerbated microvascular leakage and increased the number of acellular capillaries, while H-151 could alleviate the phenomena (Fig. 7E, F).

**Fig. 8:**
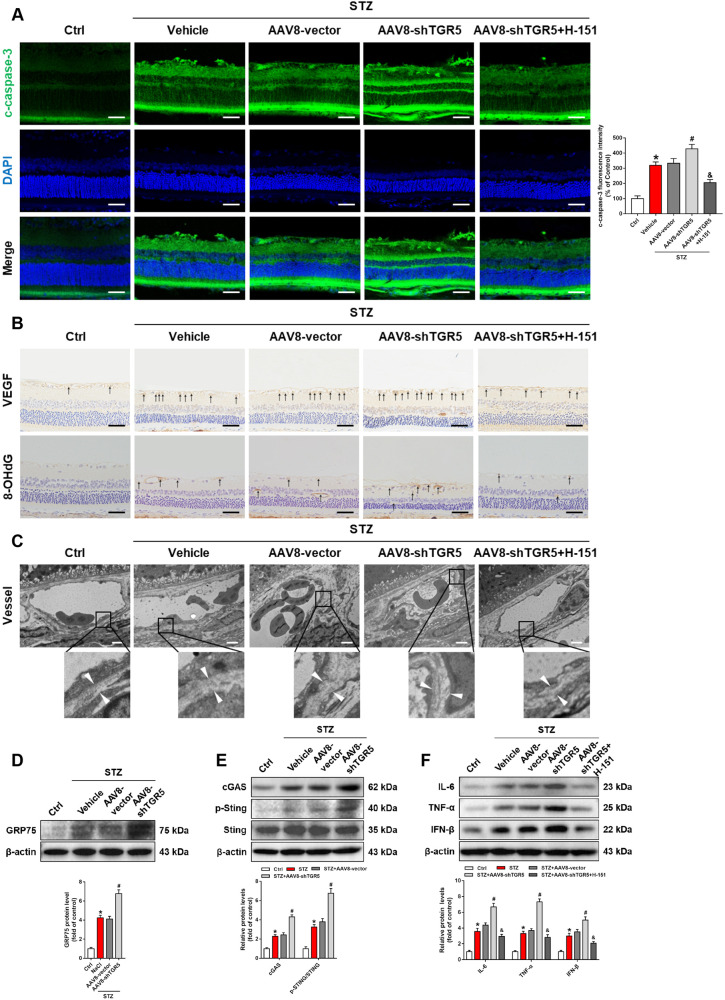
STING inhibitor ameliorates AAV-shTGR5-induced aggravation of retinal dysfunction in DR rats. **A** Representative immunofluorescence images showing expression of cleaved caspase-3 in rat retinas. Blue=DAPI. Scale bars = 50 μm. **B** Representative immunohistochemistry analysis showing VEGF and 8-OHdG expressions in rat retinal sections. Scale bars = 50 μm. **C** Representative electron microscopy images showing the thickness of basement membrane in rat retinas. Scale bars = 2 μm. **D** Representative western blot showing GRP75 protein level in rat retinas (*n* = 3). **E** Representative western blot showing cGAS, p-STING, and STING protein levels in rat retinas (*n* = 3). **F** Representative western blot showing IL-6, TNF-α, and IFN-β protein levels in rat retinas (*n* = 3). Data are shown as mean ± SD, *p < 0.05 versus Ctrl group, ^#^*p* < 0.05 versus AAV8-Vector group. ^&^*p* < 0.05 versus AAV8-shTGR5 group.

Retinal cell apoptosis is one of the factors that contribute to retinal dysfunction and DR progression [25]. We found that cleaved caspase-3 was significantly upregulated in the AAV-shTGR5 group, while H-151 treatment significantly reduced retinal cell apoptosis (Fig. 8A). STZ treatment resulted in an increase in the expression of VEGF, which was further accentuated by AAV-shTGR5. This effect was reversed by H-151 (Fig. 8B). Furthermore, 8-OHdG was reversed by H-151 (Fig. 8B). Electron microscopy revealed significantly thicker retinal capillary basement membrane in diabetic retinas compared with controls, and AAV-shTGR5 further accentuated basement membrane thickening (Fig. 8C). Next, the relationship between TGR5 and the cGAS-STING pathway was further validated. Compared with the control group, the expressions of GRP75, cGAS, and p-STING were upregulated following TGR5 silencing (Fig. 8D, E). Inflammatory factors TNF-α, IL-6, and IFN-β were also exacerbated after AAV-shRNA-mediated TGR5 silencing. In addition, the STING inhibitor reversed the inflammatory phenotypes in the AAV-shTGR5 group (Fig. 8F). In conclusion, our results suggested that TGR5 could influence the inflammatory response of Müller cells through negative regulation of cGAS-STING signaling.

## Discussion

**Fig. 9:**
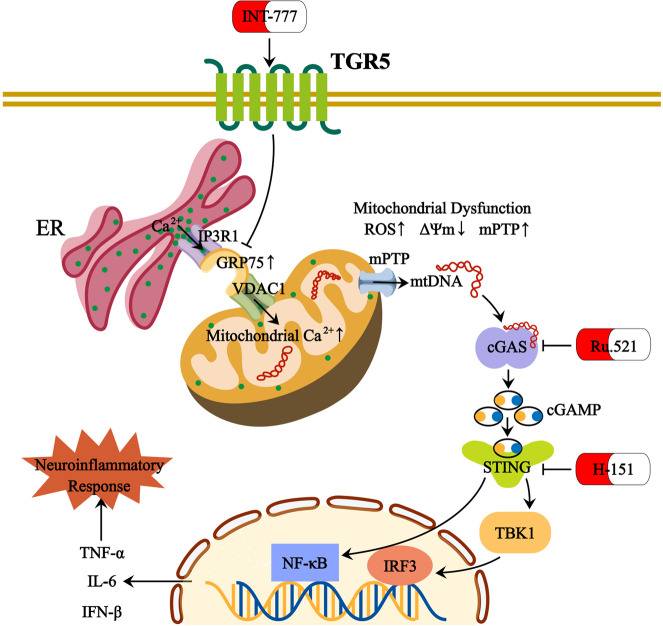
Mechanistic diagram summarizing the regulatory effect of TGR5 in mitochondrial dysfunction and neuroinflammation in Müller cells. TGR5 activation blocks GRP75-mediated MAMs formation and subsequently inhibits mitochondrial Ca^2+^ overload in Müller cells, which improves mitochondrial homeostasis and alleviates cGAS-STING pathway-mediated neuroinflammation.

The present study demonstrated that TGR5 is closely related to mitochondrial Ca^2+^ overload and the inflammatory response in Müller cells during the development of DR. The IP3R1-GRP75-VDAC axis mediated Ca^2+^ transmission from ER to mitochondria. TGR5 alleviated mitochondrial dysfunction by inhibiting ER-mitochondrial coupling through IP3R1-GRP75-VDAC axis. As a result, mtDNA release from damaged mitochondria was inhibited, and cGAS-STING-mediated inflammatory response was alleviated. The specific mechanism is illustrated in Fig. 9.

Our previous study found that HG led to changes in mitochondrial morphology and function, but the reasons for mitochondrial dysfunctions were not evident [26]. Mitochondrial dysfunction has been shown to be associated with a variety of diseases [27–29]. They play important roles in regulating the energy-generating process, redox imbalance, and Ca^2+^ homeostasis [30–32]. Mitochondrial dysfunction is chiefly manifested as abnormal energy metabolism, decreased MMP, excessive mtROS, and perturbed mitochondrial dynamics [33]. We demonstrate herein that TGR5 activation alleviates Müller cells injury by reducing HG-induced mitochondrial dysfunction.

Mitochondrial Ca^2+^ homeostasis is closely related to mitochondrial function. Under physiological states, mitochondrial Ca^2+^ promotes the tricarboxylic acid cycle (TCA cycle) and oxidative phosphorylation by increasing the activity of Ca^2+^-sensitive dehydrogenases, including pyruvate dehydrogenase phosphatase, isocitrate dehydrogenase, and α-ketoglutarate dehydrogenase [34]. However, excessive levels of mitochondrial Ca^2+^ can generate ROS and induce mPTP opening and further cause mitochondrial dysfunction [35]. Our results indicated that TGR5 activation inhibited HG-induced mitochondrial Ca^2+^ overload. BAPTA-AM, a Ca^2+^ chelator, reversed TGR5 silencing-induced mitochondrial dysfunction and cytotoxicity. These results suggest that TGR5 regulates mitochondrial function by modulating mitochondrial Ca^2+^.

ER is the main organelle for intracellular Ca^2+^ storage. The uptake and release of Ca^2+^ from ER directly affect intracellular Ca^2+^ homeostasis. ER and mitochondria are dynamically adjusted in response to the external environment. MAMs, highly specialized subcellular regions composed of ER and mitochondria, are involved in various cellular events such as Ca^2+^ homeostasis, lipid synthesis and utilization, ER stress, and mitochondrial fusion, among others [36]. Our study indicated that TGR5 blocked HG-induced MAM formation. IP3R is a Ca^2+^-regulated cation channel that allows Ca^2+^ to be effluxed from the ER to the cytoplasm. VDAC is a channel protein located in the outer mitochondrial membrane that is capable of regulating Ca^2+^ entry into the mitochondria. GRP75 links ER and mitochondria by simultaneously binding to IP3R and VDAC1 [19, 37]. Our research indicated that TGR5 reversed HG-induced abnormally high expression of GRP75. In ER, in addition to IP3R1, ryanodine receptors (RYR) may also be involved in mitochondrial calcium transport in MAM. This probably explains why the recovery of cell viability, mitochondrial function and protein expression after treatment with INT-777 is only up to 50%.

The accumulation of unfolded or misfolded proteins under pathological conditions triggers an imbalance of intracellular unfolded protein response (UPR) and induces ERS. Tunicamycin (an ERS inducer, TUN) promoted the formation of the IP3R1-GRP75-VDAC1 complex and caused cardiomyocyte apoptosis, which played an important role in diabetic atrial remodeling [22]. ERS is also involved in the pathogenesis of DR. Our results showed that TGR5 alleviated HG-, TUN-, and advanced glycation end-products (AGEs)-induced ERS (Fig. S5A–C). PERK, an ER-transmembrane protein, was found to interact with MFN2 and further stabilize MAM [38]. Interestingly, our study showed that TGR5 could suppress PERK activation (Fig. S5A–C). Therefore, we speculate that PERK also participates in TGR5-mediated MAM alterations. Future studies are warranted to determine this. Mitochondrial quality control such as mitophagy dysregulation, lysosomal dysfunction and mitogenesis deficiency may also be important in diabetic retinopathy and Müller cell dysfunction. We speculate that the inability to recover fully by TGR5 agonist INT-777 in Müller cells under HG exposure may be implicated. Mitochondrial dynamics are regulated by the balance of fission and fusion. Imbalances in fission and fusion cause mitochondrial abnormalities that lead to various adverse events [39]. Drp1 is a major regulator of mitochondrial fission. It is not randomly distributed in the cytoplasm but enriched near ER. Drp1 participates in maintaining ER-mitochondrial contacts and causes mitochondrial fission by cleaving the mitochondrial phospholipid bilayer [40]. However, it has also been documented that Drp1-mediated mitochondrial fission leads mitochondria away from ER, disrupting the ER-mitochondrial coupling in the striatum of Huntington’s disease mice [41]. We found that the TGR5 agonist rescued HG-induced mitochondrial fission by inhibiting Drp1 activation. However, it remains unclear whether Drp1 is directly related to MAMs, and further studies are required to investigate the hypotheses.

As a special form of deoxyribonucleic acid, mtDNA plays a key role in regulating OXPHOS system. Some studies have suggested that mtDNA is spatially associated with MAMs, indicating that mtDNA is associated with ER-mitochondrial coupling [42]. We confirmed that mtDNA was released from the damaged mitochondria to the cytoplasm. Subsequently, cytoplasmic mtDNA bound to cGAS, leading to the production of cGAMP. Mitochondrial dysfunction and cGAS-STING activation are closely related to retinal inflammation. Our study found that TGR5 activation inhibited MAM formation, maintained mitochondrial function, and subsequently downregulated cGAMP-mediated STING activation.

The cGAS-STING pathway is involved in various pathophysiological processes, including inflammatory responses, cellular senescence, and cancer [43]. Chronic inflammation is a feature of neurodegenerative pathology. Activation of the cGAS-STING pathway is involved in the progression of neuroinflammatory diseases [44, 45]. Recent studies have shown that the inhibition of cGAS-STING activation results in the alleviation of retinal inflammation and nerve degeneration [46, 47]. STING signaling consists of IFN- and non-IFN-dependent pathways. Upon binding to ligands, STING is dimerized and transported from the ER to the Golgi apparatus. The complex recruits and activates TBK1 and IKKε and then phosphorylates IRF3. Phosphorylated IRF3 is translocated into the nucleus and mediates the expressions of inflammatory factors (IFN-β and IL-6, etc). The IFN-independent pathway is mainly mediated by NF-κB. Phosphorylated NF-κB is translocated into the nucleus and subsequently induces the expressions of inflammatory factors (TNF-α, IL-6, and IL-1β, etc.). Müller cells are involved in the regulation of neuroinflammatory responses in the retina. They are capable of secreting proinflammatory cytokines and promoting angiogenesis in DR [48, 49]. Our results demonstrated that TGR5 suppressed STING/TBK1-mediated IRF3 and NF-κB phosphorylation, while these effects were reversed by cGAS or STING inhibition. In addition, it should be noted that STING is able to localize in the MAM microdomains and induce ERS [50]. Furthermore, it has been shown that STING can influence the dynamics of mitochondria [51]. TGR5 agonist is able to regulate STING and also influence mitochondrial fission. The causal link between them merits further exploration. Nonetheless, we found that TGR5 activation blocked cGAS-STING-mediated inflammatory response and alleviated retinal injury in DR rats. Also, AAV-TGR5-induced aggravation of retinal dysfunction was reversed by the STING inhibitor. The above results suggested that TGR5 mitigates DR progression by inhibiting the cGAS-STING pathway.

In conclusion, we found that TGR5 activation inhibited GRP75-mediated MAM formation in Müller cells, which in turn prevented mitochondrial dysfunction by inhibiting the transfer of Ca^2+^ from ER to the mitochondria and blocked neuroinflammatory responses triggered by the cGAS-STING signaling pathway. However, this study has a few shortcomings worth addressing. First, we tentatively conclude that TGR5 could downregulate GRP75 to inhibit MAM formation. Nevertheless, the specific mechanism whereby TGR5 regulates GRP75 remains to be determined. Second, our findings preliminary suggest that STING may be involved in MAM formation but we did not conduct in-depth studies on it. It may be a novel avenue for DR progression, and additional studies will be conducted in our following investigations. Despite these limitations, we believe that the present findings contribute to a better understanding of the relationships among MAMs formation, cGAS-STING activation, and neuroinflammation in DR. Our results demonstrate that TGR5 may serve as a therapeutic strategy for DR.

### Reporting summary

Further information on research design is available in the Nature Research Reporting Summary linked to this article.

## Supplementary information

Supplementary Material

Reporting Summary

## Data Availability

The data that support the findings of this study are available from the corresponding author upon reasonable request.
