# Supplementary material for: TGR5 supresses cGAS/STING pathway by inhibiting GRP75-mediated endoplasmic reticulum-mitochondrial coupling in diabetic retinopathy
Source: Cell Death Dis. 2023 Sep 1;14(9):583. doi: 10.1038/s41419-023-06111-5 (PMC10474119; doi:10.1038/s41419-023-06111-5)
Supplement: Supplementary file 1 — Supplementary Material [file 41419_2023_6111_MOESM1_ESM.docx]

SUPPLEMENTARY MATERIAL


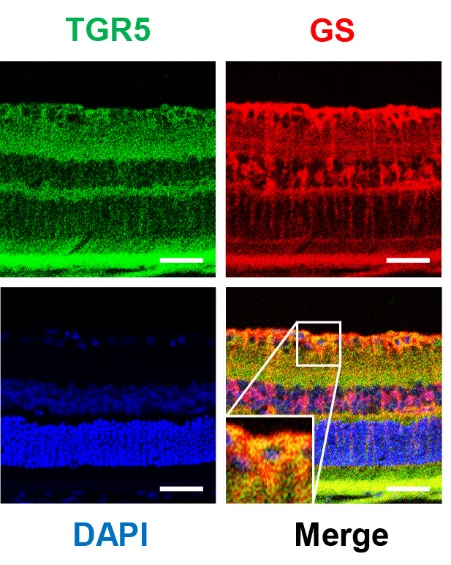


**Fig. S1.** Representative immunofluorescence images showing co-localization of TGR5 (green) and GS (red) in rat retinal sections. blue = DAPI; yellow = Merge. Scale bars = 25 μm.


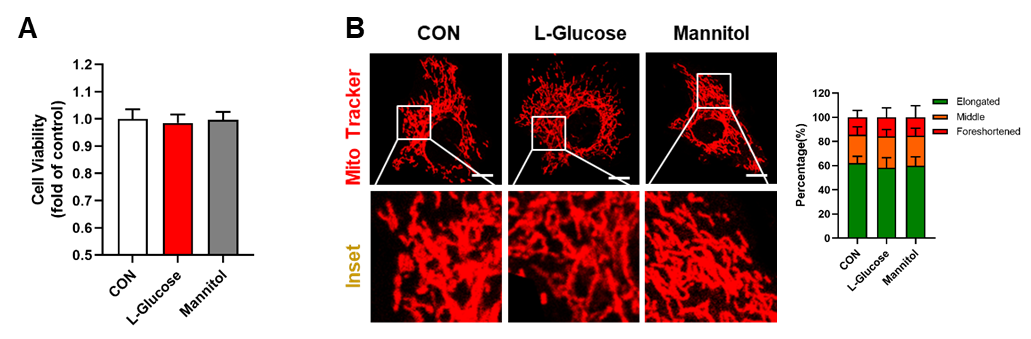


**Fig. S2.** Müller cells were treated with PBS, L-Glucose (33 mM), or Mannitol (33 mM) for 48 h in DMEM with 2% FBS. **A** Cell viability was determined by CCK-8 (*n* = 6). **B** Representative confocal images and bar graph showing mitochondrial network morphology. Scale bars = 10 μm. Data are presented as mean ± SD.


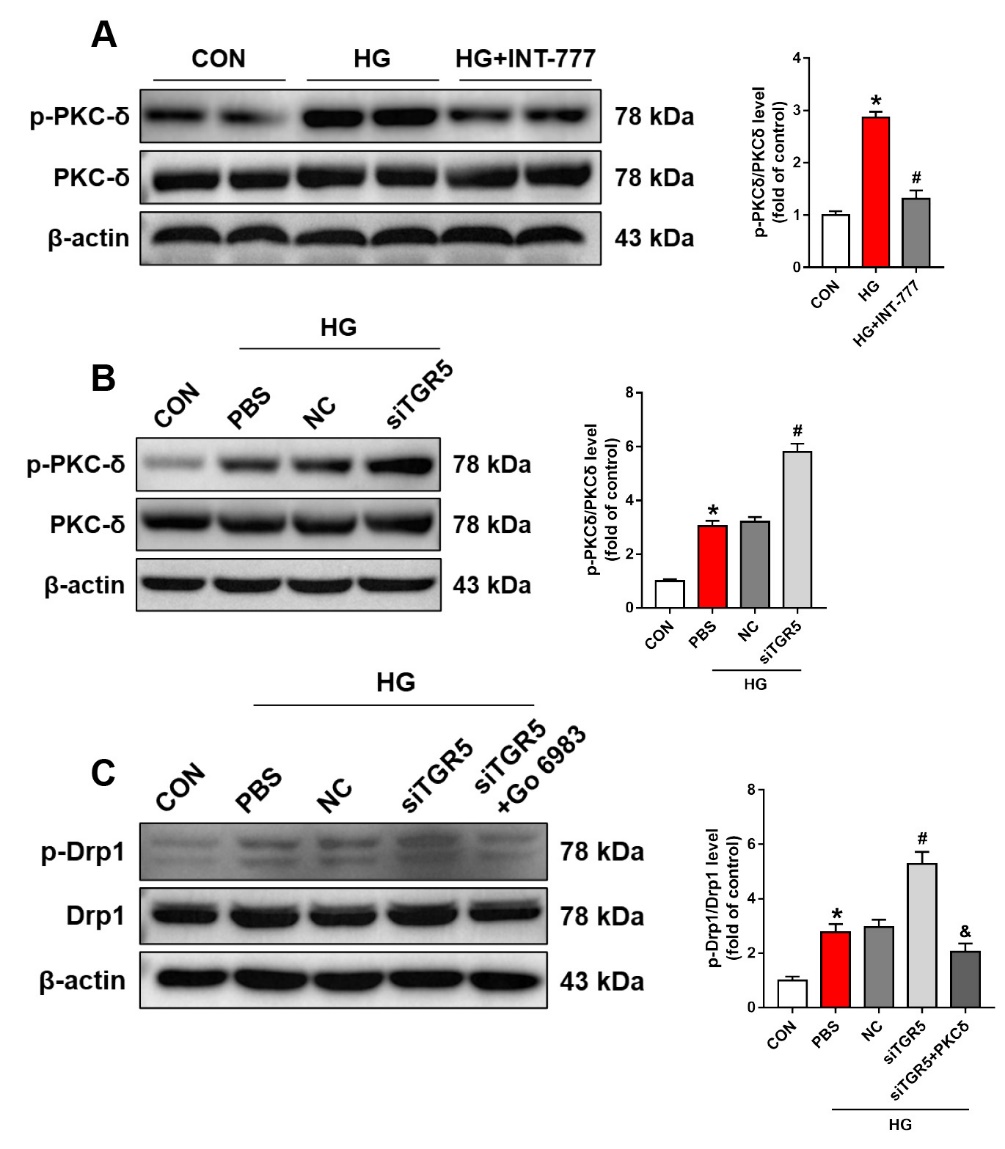


**Fig. S3.** **A** Representative Western Blot showing p-PKCδ, and PKCδ levels in Müller cells under high glucose stimulation with or without INT-777 (*n* = 3). **B** Representative Western Blot showing p-PKCδ and PKCδ levels in Müller cells which were transfected with TGR5 siRNA under high glucose stimulation (*n* = 3). **C** Representative Western Blot showing p-Drp1 and Drp1 protein expressions in Müller cells transfected with TGR5 siRNA with or without PKCδ inhibitor under high glucose stimulation (*n* = 3). Data are shown as mean ± SD, ******p* < 0.05 *vs.* control (CON) group, ^#^*p* < 0.05 *vs.* high glucose (HG) group. ^&^*p* < 0.05 *vs.* TGR5 knockdown (siTGR5) group.


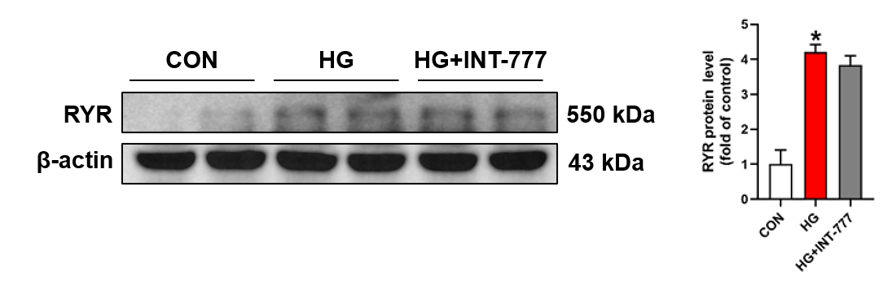


**Fig. S4.** Representative western blot showing RYR protein expression level (*n* = 3). Data are presented as mean ± SD, ******p* < 0.05 versus control (CON) group.


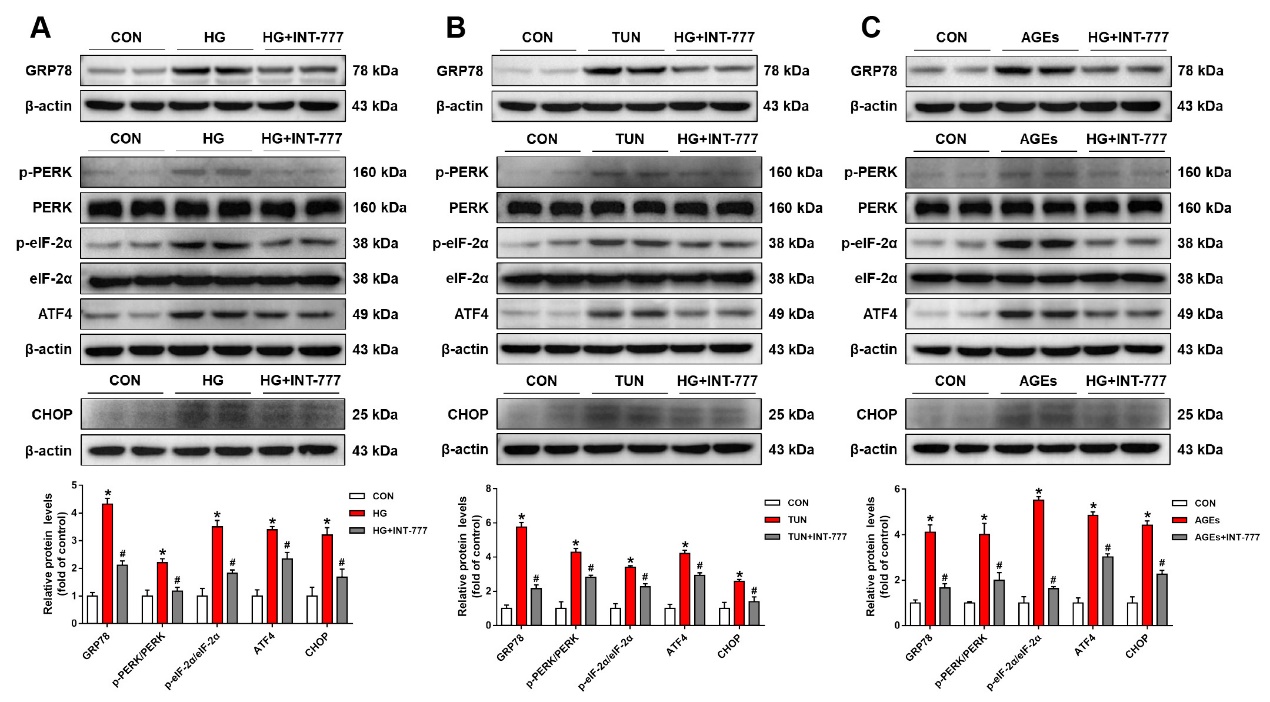


**Fig. S5. A** Representative Western Blot showing GRP78, p-PERK, PERK, p-eIF-2α, eIF-2α, ATF4, and CHOP levels in Müller cells with or without INT-777 under high glucose stimulation (*n* = 3). **B** Representative Western Blot showing GRP78, p-PERK, PERK, p-eIF-2α, eIF-2α, ATF4, and CHOP levels in Müller cells with or without INT-777 under TUN stimulation (*n* = 3). **C** Representative Western Blot showing GRP78, p-PERK, PERK, p-eIF-2α, eIF-2α, ATF4, and CHOP levels in Müller cells with or without INT-777 under AGEs stimulation (*n* = 3). Data are shown as mean ± SD, ******p* < 0.05 versus control (CON) group, ^#^*p* < 0.05 versus HG group.

**Table S1. The sequences of the siRNA**

| **Product number** | **Product name** | **Serial number** |
| --- | --- | --- |
| siG1466101050 | si-TGR5-001 | GTCTGGCATTGCCCACATT |
| siG1466101059 | si-TGR5-002 | CCTGTACCTCGAAGTCTAT |
| siG141217140319 | si-TGR5-003 | TCTACTTGGCTCCCAACTT |
| stB0006736A | si-GRP75-001 | GCGATATGATGATCCTGAA |
| stB0006736B | si-GRP75-002 | GAGTCAGATTGGAGCATTT |
| stB0006736C | si-GRP75-003 | GCTGGAATGGCCTTAGTCA |
| siB161011044323 | NC-siRNA | GGCTCTAGAAAAGCCTATGC |
| pHS-ASR-0062 | TGR5-shRNA (rat) | GGGCCTGTAACTCTGTTATCT |
| pHS-ASR-LW416 | NC shRNA(rat) | TTCTCCGAACGTGTCACGTTT |

**Table S2. Primers for Quantitative Real-Time PCR**

| Primer | Sequence |
| --- | --- |
| Human MTND1 Forward | CTCTTCGTCTGATCCGTCCT |
| Human MTNDI Reverse | TGAGGTTGCGGTCTGTTAGT |
| Human MTND2 Forward | GTAGACAGTCCCACCCTCAC |
| Human MTND2 Reverse | TTGATCCCGTTTCGTGCAAG |
| Human gDNA Forward | GTAACCCGTTGAACCCCATT |
| Human gDNA Reverse | CCATCCAATCGGTAGTAGCG |

**Table S3. Main materials and reagents**

| **Reagents** | **Source** | **Identifier** |  | | |
| --- | --- | --- | --- | --- | --- |
| Streptozotocin | Sigma-Aldrich | S0130 |  | | |
| Evans Blue | Sigma-Aldrich | E2129 |  | | |
| Glucose | Sigma-Aldrich | G8270 |  | | |
| INT-777 | MCE | HY-15677 |  | | |
| SR717 | MCE | HY-131454 |  | | |
| Ru.521 | MCE | HY-114180 |  | | |
| H-151 | MCE | HY-112693 |  | | |
| Mito-TEMPO | MCE | HY-112879 | |  |  |
| BAPTA-AM | MCE | [HY-](https://www.medchemexpress.cn/fludarabine.html)100545 | |  |  |
| Protein A/G magnetic beads | MCE | HY-K0202 | |  |  |
| Cell counting kit-8 | Beyotime | C0043 |  | | |
| JC1 assay kit | Beyotime | C2006 |  | | |
| mPTP assay kit | Beyotime | C2009S | |  |  |
| Cell mitochondria isolation kit | Beyotime | C3601 |  | | |
| Mito-Tracker Red | Beyotime | C1035 |  | | |
| Mito-Tracker Green | Beyotime | C1048 |  | | |
| ER-Tracker Green | Beyotime | C1042 |  | | |
| Penicillin-Streptomycin solution | Beyotime | C0222 |  | | |
| RIPA lysis buffer | Beyotime | P0013B |  | | |
| Rhod-2 AM | YEASEN | 40776ES72 |  | | |
| PCR mycoplasma detection kit | Shanghai Yise Medical Technology | PM008 |  | | |
| HE staining kit | BOSTER | AR1180 |  | | |
| IHC staining kit | BOSTER | SV0004 |  | | |
| PAS/Hematoxylin staining kit | Solarbio | G1281 |  | | |
| MitoSOX™ Red | Thermo Fisher | M36008 |  | | |
| DAPI | Thermo Fisher | R37606 |  | | |
| ECL kit | Thermo Fisher | 34580 |  | | |
| Goat anti-Mouse IgG (H+L), Alexa Fluor 555 | Thermo Fisher | A-21422 |  | | |
| Goat anti-Rabbit IgG (H+L), Alexa Fluor 555 | Thermo Fisher | A-21428 |  | | |
| Goat anti-Mouse IgG (H+L), Alexa Fluor 488 | Thermo Fisher | A-11001 |  | | |
| Pierce™ IP lysis buffer | Thermo Fisher | 87787 |  | | |
| 2’3’-cGAMP ELISA Kit | Cayman | 501700 |  | | |
| riboFECT CP Transfection Kit | Ribo | C10511-1 |  | | |
| TGR5 siRNA Kit | Ribo | siG1466101050 |  | | |
| GRP75 siRNA Kit | Ribo | stB0006736 |  | | |
| AAV8-shTGR5 | Vigene Biosciences | pHS-ASR-0062 |  | | |
| Goat Anti-Mouse IgG(H+L) | Jackson | 115-035-003 |  | | |
| Goat Anti-Rabbit IgG(H+L) | Jackson | 111-035-003 |  | | |
| [Fetal Bovine Serum](https://www.thermofisher.com/cn/zh/home/life-science/cell-culture/mammalian-cell-culture/fbs.html?SID=fr-fbs-main) | Gibco | 10099-141 |  | | |
| Trypsin-EDTA (0.25%) | Gibco | 25200072 |  | | |
| DMEM/F-12 | Gibco | 11330057 |  | | |

**Table S4. Primary antibodies**

| **Reagents** | **Source** | **Identifier** |  |
| --- | --- | --- | --- |
| Rabbit monoclonal anti-GRP75 | CST | #3593 | |
| Rabbit monoclonal anti-p-DRP1 | CST | #4494 |  |
| Rabbit monoclonal anti-cGAS | CST | #15102 |  |
| Rabbit monoclonal anti-p-STING | CST | #50907 |  |
| Rabbit monoclonal anti-STING | CST | #13647 |  |
| Rabbit monoclonal anti-p-TBK1 | CST | #5483 |  |
| Rabbit monoclonal anti-TBK1 | CST | #3504 |  |
| Rabbit monoclonal anti-p-NF-κB | CST | #3033 |  |
| Rabbit monoclonal anti-NF-κB | CST | #8242 |  |
| Rabbit monoclonal anti-p-IRF3 | CST | #29047 |  |
| Rabbit monoclonal anti-IRF3 | CST | #11904 |  |
| Rabbit monoclonal anti-IL-6 | CST | #12153 |  |
| Rabbit monoclonal anti-TNF-α | CST | #6945 |  |
| Rabbit monoclonal anti-IFN-β | CST | #73671 |  |
| Rabbit monoclonal anti-GRP78 | CST | #3177 |  |
| Rabbit monoclonal anti-p-PERK | CST | #3179 |  |
| Rabbit monoclonal anti-PERK | CST | #3192 |  |
| Rabbit monoclonal anti-p-eIF-2α | CST | #3398 |  |
| Rabbit monoclonal anti-eIF-2α | CST | #5324 |  |
| Rabbit monoclonal anti-ATF4 | CST | #11815 |  |
| Mouse monoclonal anti-CHOP | CST | #2895 |  |
| Rabbit polyclonal anti-TGR5 | Abcam | ab72608 |  |
| Rabbit monoclonal anti-DRP1 | Abcam | ab184247 |  |
| Rabbit monoclonal anti-p-PKCδ | Abcam | ab76181 |  |
| Rabbit monoclonal anti-PKCδ | Abcam | ab182126 |  |
| Rabbit monoclonal anti-MTCO1 | Abcam | ab203912 |  |
| Rabbit monoclonal anti-TOMM20 | Abcam | ab186735 |  |
| Mouse monoclonal anti-GS | Abcam | ab64613 | |
| Mouse monoclonal anti-IP3R1 | Santa | sc-271197 | |
| Mouse monoclonal anti-VDAC1 | Santa | sc-390996 | |
| Mouse monoclonal anti-dsDNA | Santa | sc-58749 |  |
| Mouse monoclonal anti-VEGF | Santa | sc-7269 | |
| Mouse monoclonal anti-8-OHdG | Santa | sc-393871 | |
| Mouse monoclonal anti-TOMM20 | Santa | sc-17764 | |
| Normal mouse IgG | Santa | sc-2025 |  |
| Mouse monoclonal anti-GFAP | Sigma-Aldrich | G3893 |  |
| Rabbit polyclonal anti-SDHB | Proteintech | 10620-1-AP |  |
| Rabbit polyclonal anti-NDUFB8 | Proteintech | 14794-1-AP |  |
| Rabbit polyclonal anti-ATP5A1 | Proteintech | 14676-1-AP |  |
| Rabbit polyclonal anti-UQCRC2 | Proteintech | 14742-1-AP |  |
| Mouse monoclonal anti-β-actin | Proteintech | 66009-1-Ig |  |
